# Viral control of biomass and diversity of bacterioplankton in the deep sea

**DOI:** 10.1038/s42003-020-0974-5

**Published:** 2020-05-22

**Authors:** Rui Zhang, Yanxia Li, Wei Yan, Yu Wang, Lanlan Cai, Tingwei Luo, Huifang Li, Markus G. Weinbauer, Nianzhi Jiao

**Affiliations:** 1 State Key Laboratory of Marine Environmental Science, Fujian Key Laboratory of Marine Carbon Sequestration, College of Ocean and Earth Sciences, Xiamen University (Xiang’an), 361102 Xiamen, Fujian China; 2 Department of Ocean Science, The Hong Kong University of Science and Technology, Clear Water Bay, Hong Kong China; 3 Southern Marine Science and Engineering Guangdong Laboratory (Zhuhai), 519080 Zhuhai, China; 4 Laboratoire d’Océanographie de Villefranche (LOV), UPMC, Université Paris 06, CNRS, Sorbonne Universités, 181 Chemin du Lazaret, 06230 Villefranche-sur-Mer, France

**Keywords:** Microbiology, Microbial ecology

## Abstract

Viral abundance in deep-sea environments is high. However, the biological, ecological and biogeochemical roles of viruses in the deep sea are under debate. In the present study, microcosm incubations of deep-sea bacterioplankton (2,000 m deep) with normal and reduced pressure of viral lysis were conducted in the western Pacific Ocean. We observed a negative effect of viruses on prokaryotic abundance, indicating the top-down control of bacterioplankton by virioplankton in the deep-sea. The decreased bacterial diversity and a different bacterial community structure with diluted viruses indicate that viruses are sustaining a diverse microbial community in deep-sea environments. Network analysis showed that relieving viral pressure decreased the complexity and clustering coefficients but increased the proportion of positive correlations for the potentially active bacterial community, which suggests that viruses impact deep-sea bacterioplankton interactions. Our study provides experimental evidences of the crucial role of viruses in microbial ecology and biogeochemistry in deep-sea ecosystems.

## Abstract (web summary)

Rui Zhang et al. demonstrate that reduced viral pressure decreases the diversity of deep-sea bacterioplankton in the western Pacific Ocean. This study suggests that viruses sustain a diverse microbial community in deep-sea environments.

## Introduction

Virioplankton are the most abundant biological entities not only in the surface ocean but also in the deep sea (the part of the water column below 200 m in depth)^1^. Compared with their hosts (mainly prokaryotes), the number of viral particles decreases much more slowly with depth^1^. For example, in the North Atlantic, the average abundances of viruses decreased less than 50% from the surface to 3000 m in depth while those of picoplankton decreased more than 90%^2^. However, the biological, ecological, and biogeochemical roles of virioplankton in the deep sea are still largely unknown. Based on the observation of low host abundance and activity, previous studies argued that virioplankton in the deep sea are in a state of “maintenance” and thus unlikely to infect their hosts^1^. In addition, a decoupling of viral abundance between prokaryotic abundance and other microbial parameters (e.g., prokaryotic production) has been usually recorded in deep sea^3–5^. The high abundances of viral particles in the deep sea were therefore explained by allochthonous input of viral particles from the upper ocean (e.g., via sedimenting particles) and a low viral decay rate in cold, dark deep-sea environments^1,2^. However, there is also an indication that viral abundance in the deep sea is high in areas with low particle export^3,6^.

One of the ecologically important processes mediated by virioplankton in an environment is the infection and lysis of their hosts (e.g., bacterioplankton, phytoplankton, and zooplankton). Lysis eliminates infected hosts while stimulating the growth of noninfected hosts, thus altering the general diversity and community composition of microbial populations^7^. A number of studies have demonstrated the influence of viruses on bacterial communities and therefore on the function of the microbial loop in the surface ocean^8–10^. Recently, the measurements of viral production in the Atlantic Ocean, Pacific Ocean, and Mediterranean Sea^3,4,6,11,12^ indicate that virioplankton are active players in the deep-sea ecosystem and in biogeochemical cycling. At depths below 1000 m in the North Atlantic Ocean, lytic viral production varied from 0.50 to 9.55 × 10^8^ L^−1^ d^−1^^3,6^. A similar viral production rate was observed in a study performed in the western Pacific Ocean^4^. On average, 13.4% of deep-sea prokaryotic mortality was contributed by viruses in the bathypelagic waters of the Mediterranean Sea^12^. In deep-sea sediments, viruses can be the main cause of prokaryotic mortality and can greatly impact the ecology and biogeochemistry of benthic deep-sea ecosystems^13^. In surface waters, it has been demonstrated that viruses can shape host biomass and diversity; however, direct experimental evidence is lacking to show the viral impacts on prokaryotic biomass, diversity, and community structure in the deep sea.

If viruses in the deep sea exert a similar top-down control, mirroring the surface oligotrophic gyre environment, the bacterioplankton are expected to be dominated by defense specialists against viral infection. In parallel, one may expect that the fast growing opportunistic competition specialists will bloom once the top-down control by viruses is relieved. To verify this hypothesis, we used filtration and dilution techniques in microcosms to reduce the pressure of viral lysis to study the impact of virioplankton on bacterioplankton in the western Pacific deep sea (depth 2000 m). Our results showed a negative effect of viruses on prokaryotic abundance. Decreased bacterial diversity and a differing bacterial community structures in treatments with diluted viruses showed the importance of viruses for shaping bacterial diversity and sustaining a highly diverse bacterial community in the deep sea. Our study provides the experimental demonstration of the impacts of active virioplankton on host communities in deep-sea ecosystems.

## Results

**Fig. 1:**
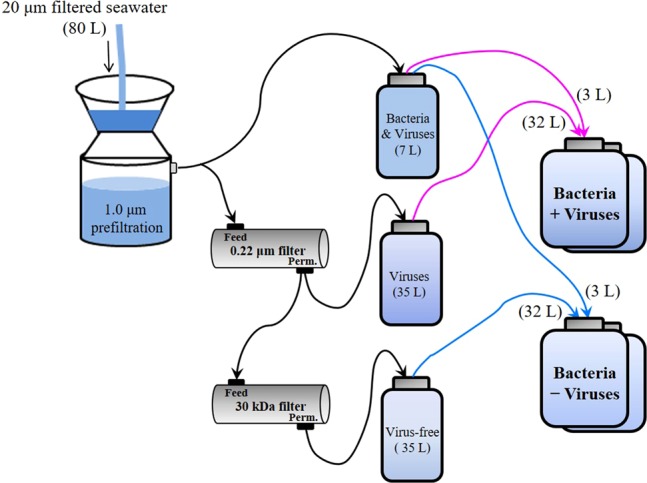
Schematic of experimental setup used in this study. Schematic diagram of the experimental setup for deep sea bacterioplankton with normal and reduced pressure of viral lysis.

**Fig. 2:**
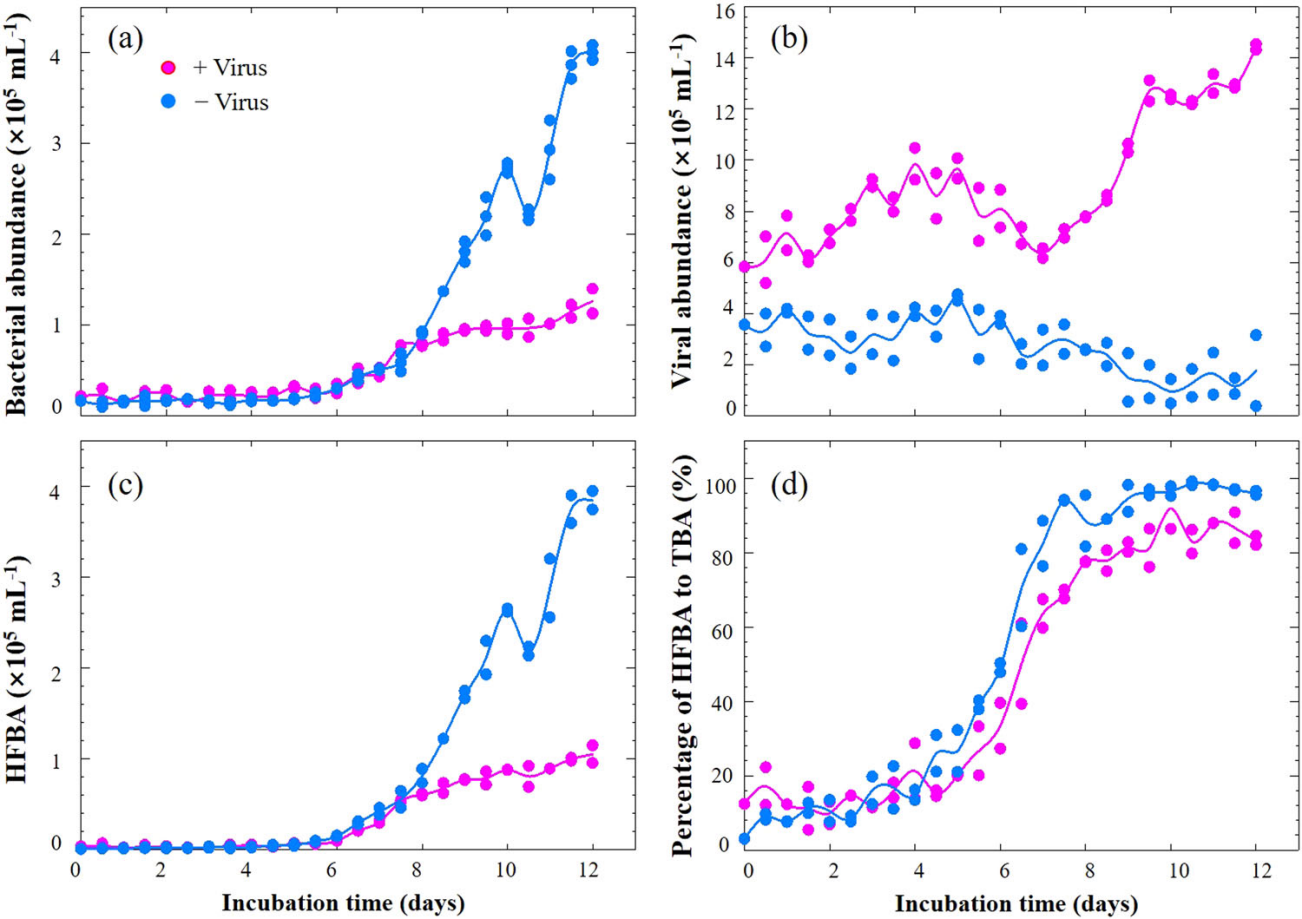
Dynamics of microbial abundance during microcosm incubation. **a** Bacterial abundance, **b** Viral abundance, **c** High-fluorescence bacterial abundance, **d** Percentages of high-fluorescence bacterial abundance to total bacterial abundance. HFBA high-fluorescence bacterial abundance, TBA total bacterial abundance. Dots show two replicates at each time point and lines represent the average values.

The microcosm experiments were conducted with 2000 m deep water samples from the western Pacific Ocean, a typical oligotrophic gyre (Fig. 1 and Supplementary Fig. 1). The in situ prokaryotic and viral abundances decreased from 0.70 and 6.17 × 10^6^ particles mL^−1^ in surface waters to 0.49 and 1.70 × 10^6^ particles mL^−1^ at 2000 m. With a series of filtrations and dilutions of water from 2000 m, microcosms were set up with the prokaryotic community either exposed to viruses (+virus) or with a reduced viral abundance (−virus) (Fig. 1). In order to investigate the development of deep sea bacterioplankton and avoid the possible limitation for bacterial growth which usually occurred in in situ deep sea environments (e.g., see refs. ^1,14^), prokaryotes were diluted at the beginning of the incubation. The initial prokaryotic abundances were 1.5 × 10^4^ particles mL^−1^ and 2.1 × 10^4^ particles mL^−1^ for −virus and +virus treatments, respectively (Fig. 2a, Supplementary Data 1). Although the filtration and dilution steps may produce slight differences between treatments (e.g., the amount of organic matter), reducing the bacterioplankton population size in our experimental setup should have alleviated this influence, and organic matter was likely not a limiting factor in our incubation system^15–17^. Resource competition among the bacteria would also have been relieved by dilution, as fewer bacteria would have been competing for the same amount of nutrients. For the −virus treatments, viral abundance was reduced to 3.55 × 10^5^ particles mL^−1^ at the beginning of incubation and showed small variations during incubation (Fig. 2b). After 12 days of incubation, viral abundance in the +virus treatments were higher and showed clear fluctuations, increasing from 5.83 × 10^5^ particles mL^−1^ to 1.44 × 10^6^ particles mL^−1^ (Fig. 2b). The virus-to-bacteria ratios in both treatments displayed similar temporal patterns and decreased after 5–7 days of incubation (Supplementary Fig. 2), which was caused by bacterioplankton growth in the −virus treatments and viral decay in the +virus treatments, respectively. Higher virus-to-bacteria ratios were always observed in the +virus treatments throughout the incubation, demonstrating the successful experimental setup with relatively strong top-down control by viruses of deep-sea bacterioplankton. In addition, compared with similar experiments for surface bacterioplankton and virioplankton^10^, a longer incubation time (12 days) was required for the deep-sea bacterioplankton and virioplankton in our study. This is consistent with the relatively low bacterial and viral activity, and then longer bacterial and viral turnover time, recorded in deep sea^1^. For example, the viral turnover times in the deep North Atlantic Ocean were 11–39 days and the turnover times of the prokaryotic community were about 34–54 days^2–4,6^.

### Effects of viruses on deep-sea bacterioplankton abundance

The bacterial abundances in the +virus and −virus treatments showed a rapid growth between Days 5 and 7, raising to 0.48 × 10^5^ particles mL^−1^ and 0.51 × 10^5^ particles mL^−1^, respectively (Fig. 2a). Afterwards, the −virus treatments were characterized by more rapid growth of bacterioplankton, reaching a higher abundance, than the +virus treatments (Fig. 2a). After 10–12 days of incubation, the bacterioplankton abundance in the –virus treatments was 3.2 times higher than that in the +virus treatments (Fig. 2a). Concurrently, the abundance of viruses in the two treatments showed distinct trends: viral abundance increased in the +virus incubations but decreased slightly in the −virus incubations (Fig. 2b). The repressing effects on prokaryotic abundance in the +virus treatments demonstrated the presence of top-down control by viruses of deep-sea bacterioplankton. The reduction of viral abundance in the −virus treatment suggests decay of viral particles due to reduced encounter rates with hosts, hence resulting in lower viral infection rates.

**Fig. 3:**
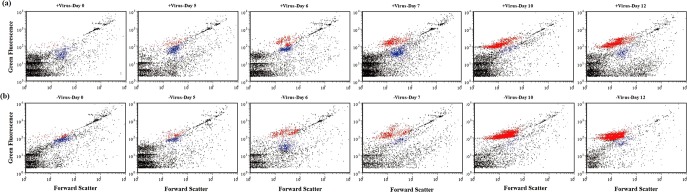
Distinct deep sea bacterioplankton populations developed with different pressure of virus lysis. **a** Flow cytograms showing development of bacterioplankton with normal viral lysis pressure. **b** Flow cytograms showing development of bacterioplankton with reduced viral lysis pressure. Red: high-DNA bacteria; Blue: low-DNA bacteria.

Since it has been proposed that viruses mainly infect active hosts, e.g., cells with high RNA content^18^, we investigated the dynamics of high-fluorescence signal bacterioplankton, containing relatively high nucleic acids^19,20^, using flow cytometry (Fig. 3). For both treatments, the abundances of high-fluorescence bacterioplankton remained relatively stable until Day 6. But in the latter period (i.e., after Day 6), high-fluorescence bacterial abundance (HFBA) increased more rapidly in the −virus treatments than in the +virus treatments were observed in the latter period (Fig. 2c). For both treatments, the percentages of high-fluorescence bacterial to total bacterial abundance (TBA) ranged from 3.0 to 49.0% for the first half of the incubation and then exceeded more than 70% during the second half. The increasing trends of HFBA were similar to those of TBA, suggesting that the growth and development of potentially active bacterial assemblages in our incubation were mainly due to the cells containing high nucleic acids content. Lower percentages of high-fluorescence cells were observed in the +virus than in the −virus treatments, especially in the latter period (Fig. 2d). The repression of active cells caused by the presence of viruses accounted for most (70%) of the difference between the abundance of total bacterioplankton in the two treatments. Our finding suggests that viruses infect and lyse active bacterioplankton cells in the deep sea, which is consistent with observations of surface water^18^. In contrast, potentially less active bacterioplankton as indicated by low nucleic acid content might be less susceptible to viral infection or the infection processes are much slower^21^. Therefore, in the deep-sea environments, the majority of bacterioplankton might be slow growing defense strategists, while the potential winners of competition for nutrients would be controlled by viruses.

### Effects of viruses on deep-sea bacterial diversity and community structure

**Fig. 4:**
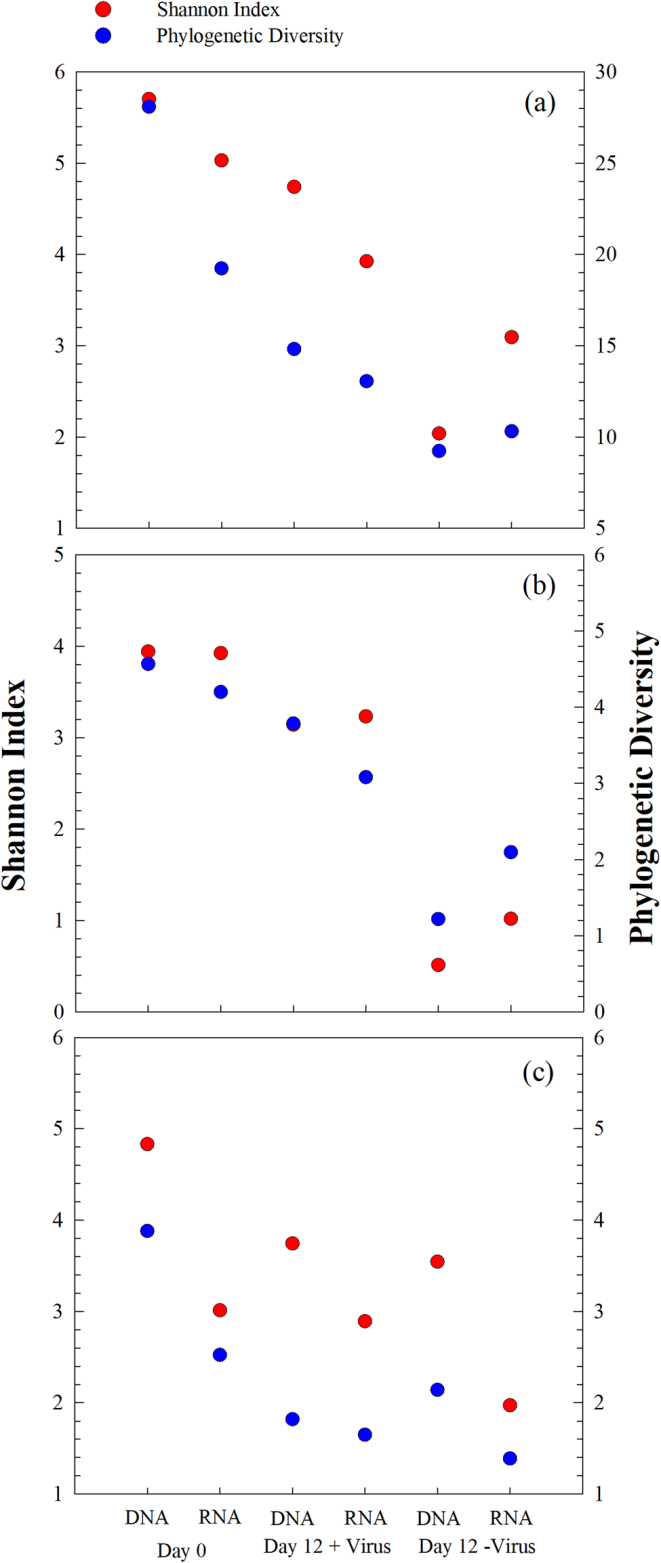
Effects of viruses on diversity of deep sea bacterioplankton. **a** Total and active bacterial diversity, **b** Total and active Alphaproteobacterial diversity, **c** Total and active Gammaproteobacterial diversity. Diversity is shown as Shannon index and phylogenetic diversity. Data show the mean of two independent incubations.

Bacterial diversity and community composition were investigated with 454 sequencing of PCR-amplified 16S rRNA genes at the DNA and RNA levels, respectively. After quality control and normalization, 64,427 sequences were obtained, and 975 OTUs were assigned based on 97% similarity of sequences. At the beginning of the incubation, the bacterial diversity (Shannon index) at the DNA level was 5.7. After 12 days of incubation, bacterial diversity decreased to 4.7 in the +virus treatments and to 2.0 in the −virus treatments (Fig. 4a). Similarly, the bacterial diversity revealed by RNA analysis decreased after incubation and was higher in the +virus treatments (Shannon: 3.9, Phylogenetic Diversity: 13.1) than in the −virus treatments (Shannon: 3.1, Phylogenetic Diversity: 10.3). Although rRNA is not simply related to bacterial activity, it has been used as tentative indicator for the active bacterial community or for an anticipatory life strategy, i.e., being able to respond quickly to environmental changes (e.g., see ref. ^22^). For the deep-sea, such environmental changes could include the mixing of water masses^23^, sinking particles, or the in situ formation of particles (e.g., see ref. ^14^).

For the major phyla present at the beginning of the experiment (e.g., Alphaproteobacteria and Gammaproteobacteria), diversity generally decreased after incubation; moreover, higher diversity was always observed for the +virus treatment than for the –virus treatment at both the DNA and RNA levels (Fig. 4b, c). Previous studies have suggested that the presence of viruses supports higher bacterial diversity in surface oceans^8,10^. The mechanism behind this phenomenon could be explained by the “kill the winner” hypothesis, which states that viruses infect and lyse the bacterial winner of resource competition, prevent their blooming, and create niches for other bacteria^24–26^. This would allow for more bacterial species to be exit in a specific environment, leading to higher diversity. Furthermore, the release of organic matter during cell lysis alters the composition and bioavailability of organic nutrients, thus changing the composition of the bacterial community in surface waters^27^. Our study suggests that viruses play a similar ecological role in structuring the microbial community in deep-sea ecosystems.

At the beginning of the incubation, the RNA-based bacterial diversity was lower than the DNA-based, indicating that some bacterial lineages were inactive (or not anticipatory) in the original bacterioplankton community. In the Day 0 samples, Alphaproteobacteria showed relatively higher diversity at the RNA level than at the DNA level (Fig. 4b), suggesting that they are highly active in situ. The loss of Gammaproteobacteria diversity was greater than that of Alphaproteobacteria after incubation with viruses (Fig. 4b, c), contributing to the majority of diversity loss of total bacterioplankton, especially at the DNA level. Similar to the total bacterial population, higher diversity loss was observed for both Alphaproteobacteria and Gammaproteobacteria in the −virus treatments than in the +virus treatments. In addition, higher diversity of active bacteria compared with that of total bacteria was observed in the −virus treatments, which was due to the same trend occurring in Alphaproteobacteria (Fig. 4a, b).

**Fig. 5:**
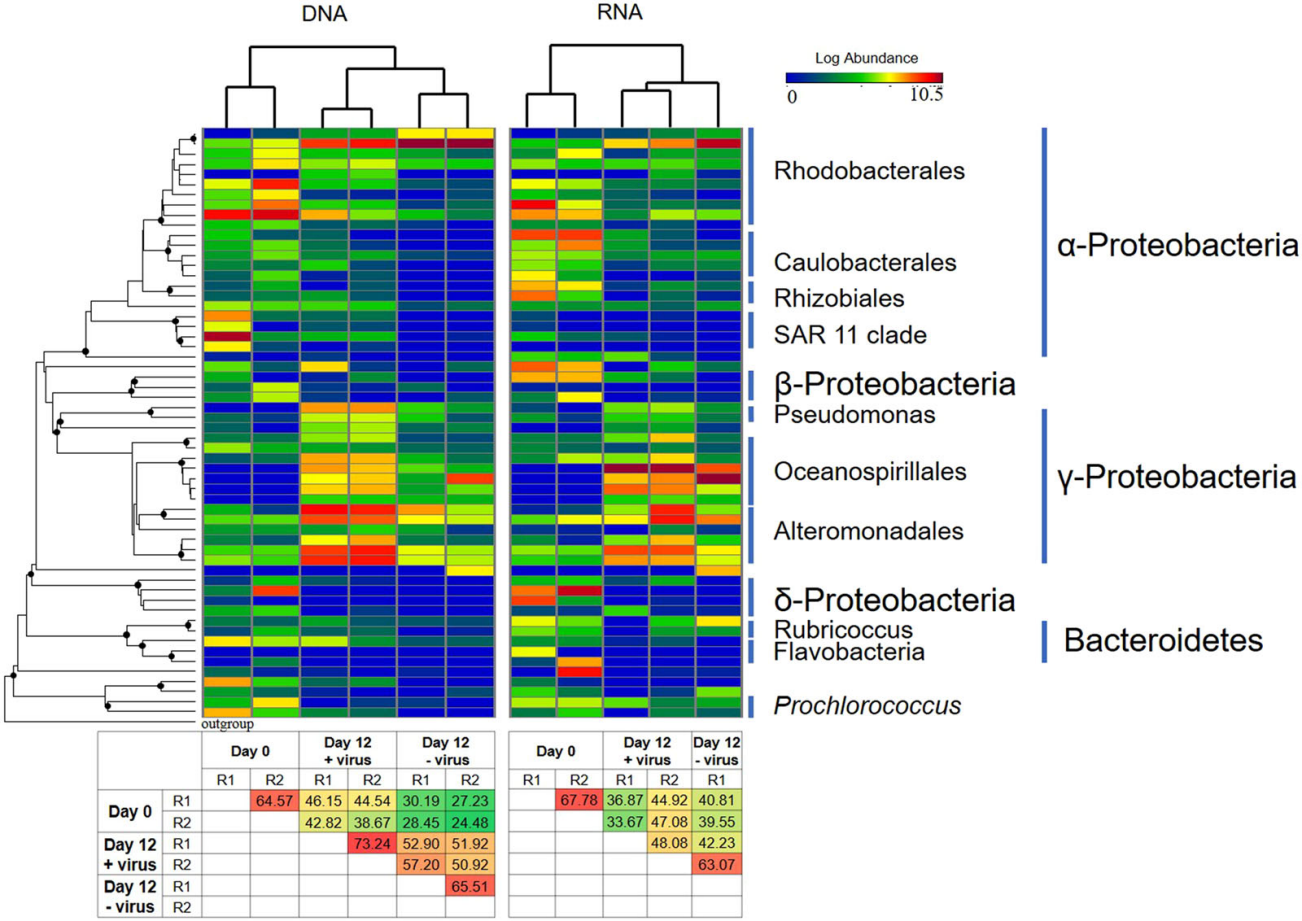
Heatmap showing the relative abundances and activity of the top 58 OTUs across the samples. The cluster analysis of bacterial community structure was generated based on Bray-Curtis distances between samples. Heatmap shows the relative abundance (log [relative abundance + 1]) for each OTU. Phylogenetic tree (on left) was reconstructed by representative sequence from each OTU with Maximum-Likelihood method using FastTree. Filled circle on the nodes show bootstrap values higher than 50% (1000 resamplings). One archaeal sequence from *Picrophilus torridus* (AE017261) was used as outgroup. The two heatmaps on the bottom show the similarity (%) of Bray–Curtis distances between samples. Day 0 samples were collected before aliquoting into microcosms and considered as control for both +virus and −virus treatments.

To represent the major bacterial lineages across all samples, the 58 most abundant OTUs (accounting for 90% of all sequences after normalization) were selected. These major OTUs were affiliated with Proteobacteria, Bacteroidetes, and Cyanobacteria (Fig. 5). Cluster analysis showed that the +virus treatments displayed more similarity to bacterial communities in the −virus treatments than those in situ (Day 0 samples) (Fig. 5). However, at both the DNA and RNA levels, the similarities between the +virus treatments and in situ environment were higher than those between the −virus treatments and in situ environment (Fig. 5). This indicates that in addition to general influences on diversity, viruses also sustain the community structure of deep-sea bacterioplankton. Similar trends have been recorded in studies on surface water (e.g., see refs. ^10,28^).

### Effects of viruses on the relative contribution of deep-sea bacterial lineages

**Fig. 6:**
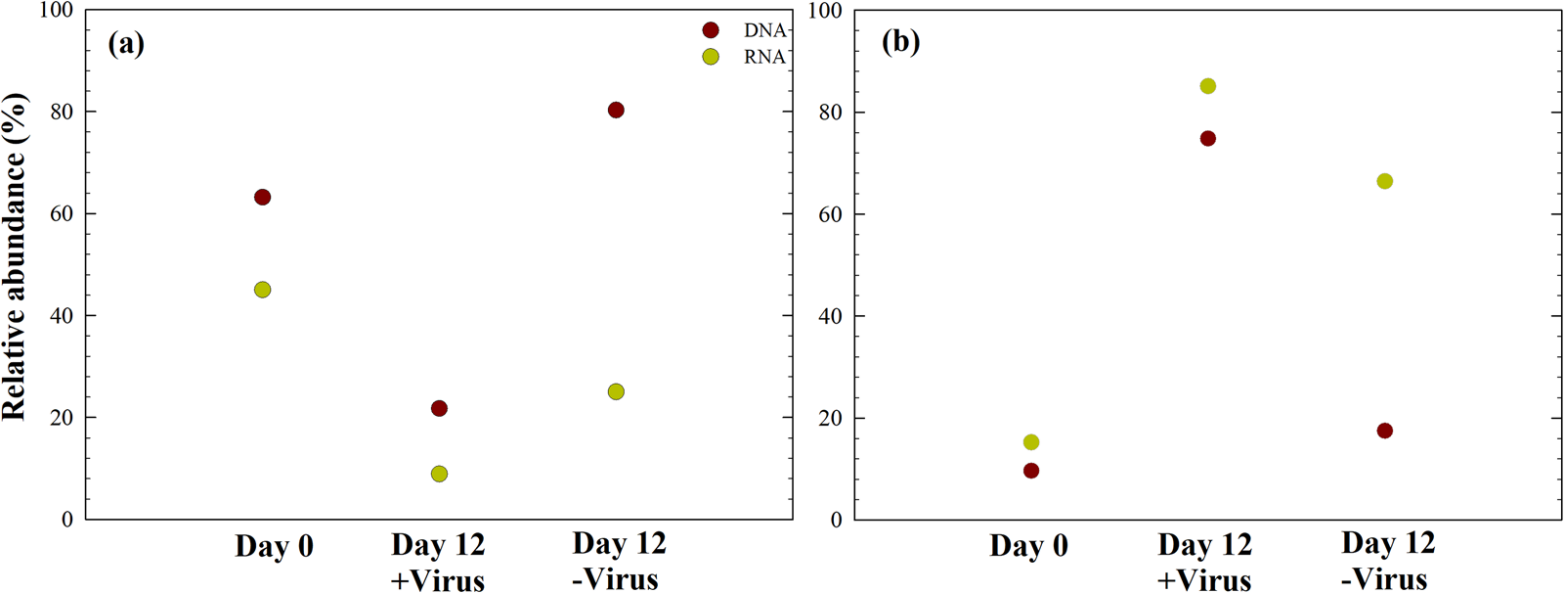
Effects of viruses on the relative abundance of two major classes of bacteria. **a** Total and active Alphaproteobacteria, **b** Total and active Gammaproteobacteria. Data show the mean of two independent incubations.

The relative abundances of specific bacterial lineages (DNA level) and potentially active bacterial lineages (RNA level) were assessed. Before incubation, Alphaproteobacteria dominated the bacterial population at both the DNA level and RNA level (Fig. 6). After 12 days of incubation with viruses, Gammaproteobacteria dominated the potentially active bacterial community (rRNA level). Detailed examination of the bacterial taxa in our experiments showed that nine of the top ten abundant OTUs showed a difference between the +virus and −virus DNA libraries, including *Sulfitobacter*, *Pseudoalteromonas*, *Marinobacter*, *Alteromonas*, *Pseudomonas*, *Oleispira*, *Oceanobacter*, and *Oceaniserpentilla* (Supplementary Data 2). This indicates that viruses have potentially strong effects on the majority of deep-sea bacterioplankton. Alphaproteobacteria (e.g., SAR11 Clade Ia, *Thalassococcus*, *Paracoccus*) were the dominant groups in the in situ bacterioplankton community. In the +virus treatment, the abundance and activity of Alphaproteobacteria were repressed compared with the −virus treatment, resulting in a population shift to Gammaproteobacteria accounting for approximately 80% of total and active bacterioplankton. When viral pressure was relieved in the −virus treatment, Alphaproteobacteria dominating the original environments retained their dominant status. These bacteria may be comparatively inactive because of the differing conditions between the in situ and the microcosm environments. In the −virus treatment, Gammaproteobacteria and some Alphaproteobacteria (e.g., *Sulfitobacter*) could have outcompeted other bacterioplankton in terms of nutrient utilization or adaption to environmental changes, thus dominating the active bacterioplankton. Such a pattern, i.e., that numerically not dominating but potentially highly active bacteria are controlled by viruses, has been demonstrated for surface water^29^. In addition, the relative abundances of Bacteroidetes and Actinobacteria decreased after incubation, and the treatments with and without the pressure of virus lysis did not differ significantly, which suggest that these bacterial groups may be less susceptible to viral infection (Supplementary Fig. 3).

### Effects of viruses on the interactions among deep-sea bacteria

**Fig. 7:**
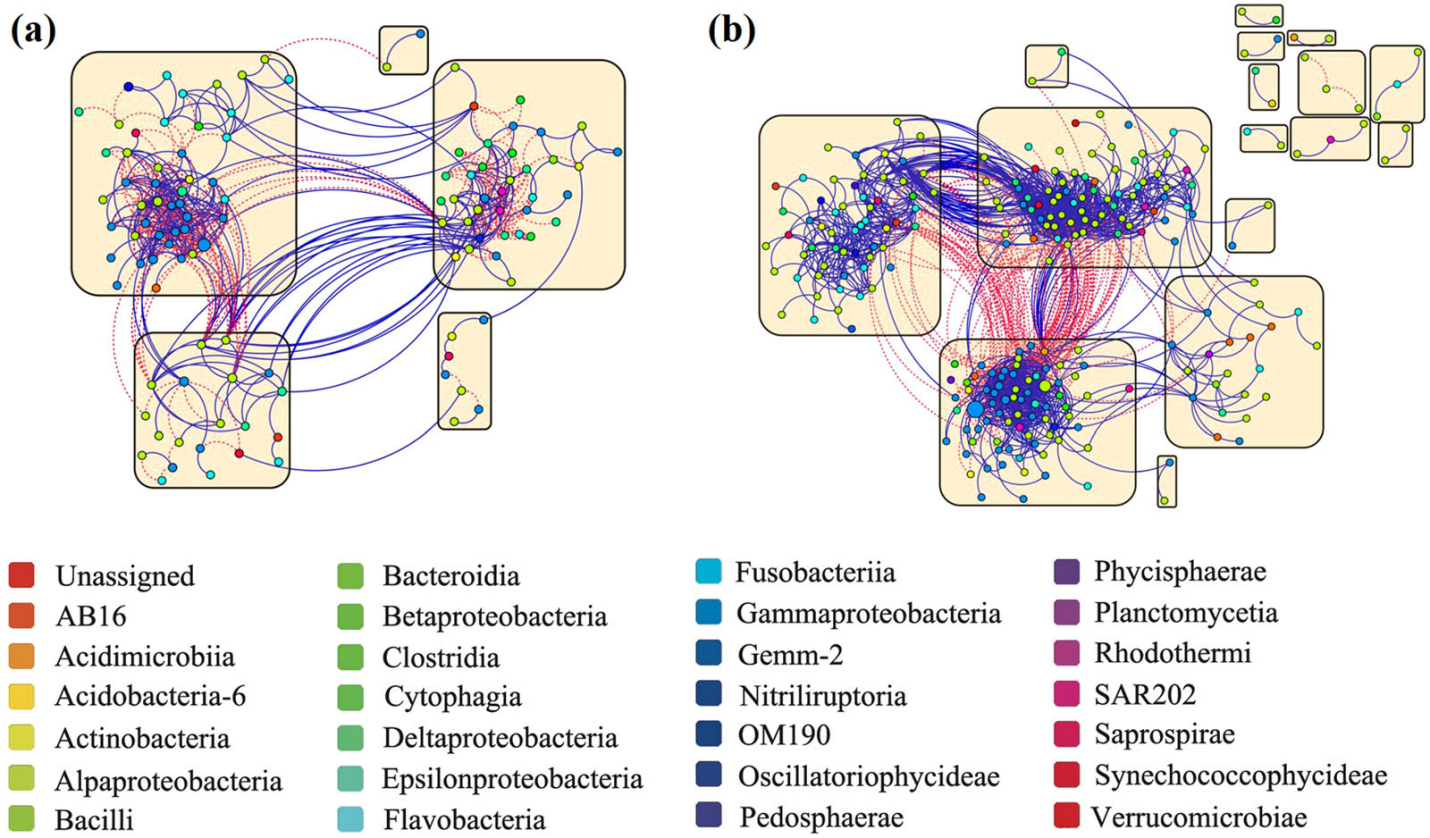
Connections among active bacteria with different pressure of virus lysis. **a** Co-occurrence network graph of bacterioplankton with normal viral lysis pressure. **b** Co-occurrence network graph of bacterioplankton with reduced viral lysis pressure. The nodes, representing OTUs, are colored according to phylogenetic taxa. Node size is proportional to the relative abundance of OTU. Blue and red lines indicate positive and negative connections, respectively, between nodes. Yellow squares show modules in the network.

To evaluate whether the presence or absence of viruses may have affected the active interactions among deep-sea bacterioplankton during the 12-day incubation, we constructed co-occurrence networks for RNA-inferred bacterial communities (Fig. 7 and Supplementary Fig. 4; Table 1 and Supplementary Data 3), assuming that RNA rather than DNA reflects the active community. In network analysis, the topological indices mathematically describe the microbial interactions, including degree, number of nodes, density, and clustering coefficient^30^. The density, the proportion of actual connections to the potential connections in a network, is higher in the +virus treatment than in the −virus treatment. This indicates that more bacteria are connected and a more complex network when viruses were present (Table 1). Similarly, the higher clustering coefficient of +virus treatment than −virus treatment reveals the closer connection among the bacteria with the occurrence of virus (Table 1). The more complexity of microbial community shown by network density and clustering coefficient is consistent with the higher bacterial diversity found in the +virus treatments (Fig. 4). This could be explained by the concept of “kill the winner”^24^ that viruses control competitive dominates for nutrients and allow for the co-existence of more diverse bacterial community. Previous studies have shown that abiotic and biotic pressures might increase the network complexity of microbial communities^31^. Our data demonstrate that top-down pressure from viral lysis could have similar ecological influences. A positive correlation between two nodes in the microbial network is usually explained as a mutualism or cooperation relationship, while theoretically, this can also be produced by co-infection of different hosts by the same viruses. In our incubation, a lower proportion of positive correlations was observed in the +virus bacterial community compared with the −virus bacterial community (ca. 56% vs. 85%) (Table 1), suggesting that the positive correlation of bacteria may not be the direct result of bacterial mortality induced by viral lysis. This is reasonable as viral infection is always host-specific and their host range is narrow. Also, viral lysis does not only cause mortality of hosts (hence influence competition among bacteria), but also causes a release of the cell content and cell wall content (lysis products) which can be used by bacterioplankton (“viral shunt”). This supposedly “lubricates” the microbial food web and could result in positive correlations of the network analysis.

**Table 1:** Properties of bacterial communities’ phylogenetic network at the RNA level with normal and reduced viral lysis pressure.

	Node #^a^	Link #	Density	Clustering coefficients	Total P link #	P link # within module	P link # between module	Total N link #	N link # within module	N link # between module	Modularity
+virus	112	536	0.086	0.533	300	259	41	236	201	35	0.584
−virus	256	1504	0.064	0.368	1273	1169	104	231	104	127	0.674

^a^Node # and link # represent the node number and the connection number in each network, respectively. Density refers to the proportion of actual connections to the potential connections in a network. Higher density means a more complex network. The clustering coefficient describes the proportion of closed triplets to the total number of triplets. A clustering coefficient close to 0 means that there are hardly any connections with neighbors, while a value of 1 means a node is fully connected to its neighbors. P link # and N link # represent the number of positive and negative connections between nodes in each network, respectively.

Four large modules contained more than five nodes were observed in each of +virus and −virus treatments, showing an evident modular architecture for both networks (Table 1). Since the OTUs within a module might occupy a similar ecological niche, a higher modularity value and module number observed in the −virus treatment (Fig. 7) indicated that number of niches increased without virus. In addition, the presence of virus stimulated the connections among bacteria within the module, which was evidenced by the higher clustering coefficients and increased proportion of links within modules in the +virus treatment (Table 1 and Fig. 7). Thus, the presence of virus might facilitate the stability of the niches by increasing the interactions among the bacteria in each niche. We also measured the proportions of Alphaproteobacteria and Gammaproteobacteria (the two major bacterial groups in the present study) in the networks (Supplementary Data 3). The Alphaproteobacteria to Gammaproteobacteria ratio was higher in the +virus treatments than in the −virus treatments, and the proportion of positive correlations for Gammaproteobacteria was greater than that for Alphaproteobacteria. Our results suggest that the removal of viruses might change the connection type (positive or negative) among bacteria, especially the Gammaproteobacteria. Thus, viruses could play a fundamental role in sustaining the deep-sea ecosystem by shaping the interactions among bacteria.

### Methodological considerations

In our study, we used onboard microcosm incubation (20 L) in the dark at the in situ temperature to represent deep-sea environments. A main difference between our incubation and the in situ deep-sea environment was pressure. The inactive deep-sea microbial community (e.g., allochthonous and piezosensitive) may become dominant while piezophilic and hyperpiezophilic microorganisms may disappear when incubated under atmospheric pressure^32^. However, there are evidences that pressure did not significantly affect viral activities and, subsequently, the impact of viruses on their host population^13,33^. In addition, the enclosure of microbial plankton communities may be biased by “bottle effects”, which may either stimulate the growth or enhance the loss of different planktonic groups^34,35^. However, if we assume that the “bottle effects” and effects of pressure changes in the +virus and −virus treatments were similar (as all incubation conditions were the same), differences in the ecological characteristics of bacterioplankton (Figs. 2–7) in our study should be due to the presence or absence of the top-down pressure of viral lysis. For example, the replacement of Alphaproteobacteria by Gammaproteobacteria has been frequently observed in microcosm incubations of bacterioplankton, and this was usually explained as resulting from the fast growth rate and opportunistic life strategy of Gammaproteobacteria in bottle incubations^36,37^. In our study, however, the simultaneous incubation in the −virus treatment showed a contrasting pattern: Alphaproteobacteria predominated in the DNA library, while Gammaproteobacteria predominated in the RNA library. This difference clearly indicates that viruses can also play a major role in shaping the bacterial community structure in the deep sea. In addition, the PCR-based method was applied to estimate bacterial diversity and relative abundance. Although we controlled the PCR cycle numbers and performed triplicate amplification for each sample to minimize any possible bias introduced by PCR, the relative abundance estimation may be biased by the variation of 16S rRNA gene copy number in the deep-sea bacterial genome (e.g., see ref. ^38^).

## Discussion

The deep ocean contains approximately 75% of the total pelagic prokaryotic biomass^1^. The diversity of bacterioplankton (including Bacteria and Archaea) in the deep ocean is almost as high as that in the upper ocean^1,39^. Also, the variability of microbial and viral communities on a seasonal scale is almost as high in deep water as in surface water^40^, thus supporting the idea that deep-sea environments are more dynamic than previously thought. However, the mechanisms controlling population size and diversity of deep sea bacterioplankton remain unclear. The relatively low concentration and bioavailability of dissolved organic matter in deep-sea environments lead to the assumption of bottom-up control of the population size of bacterioplankton^1^. The measured viral production rates are relatively low compared with those obtained in the surface and upper ocean^3,4,6,11,12^. Deep-sea virioplankton have thus been regarded as a comparatively inactive component. However, the few vertical investigations of lysis and grazing suggest that in deep waters, viral lysis might be more important than grazing in terms of relative contribution to the mortality of prokaryotes (e.g., see ref. ^16^). Additionally, an inverse relationship between grazing and lysis usually appeared with variations in community structure and system productivity (e.g., see ref. ^16^). These observations suggest that in the deep sea, where grazing is potentially limited, viral lysis could be a major top-down control of prokaryotes. Such a viral impact has also been demonstrated for deep-sea sediments^13^. Our experiment showed that viral communities likely exert a strong control on the population size and community structure of prokaryotes in the deep sea and may play more active roles in the dark ocean than previously thought.

Almost half of the heterotrophic prokaryotic production in the ocean occurs in mesopelagic and bathypelagic waters^1^. However, there is an obvious imbalance between the metabolic activity and the supply of organic carbon (including sinking particular organic carbon and chemolithoautotrophic production) in the deep ocean^14,41^. Based on our study and recent evidence of deep-sea viral activity^3,4,11,12^, we propose that viral cycling may account for at least some of the imbalance. Viral lysis shunts organic carbon back to the dissolved organic carbon pool, which can be used by prokaryotes, increase the supply of “bottom-up” resources, and thus, support prokaryote activity (e.g., see ref. ^13^). This may help to solve the paradox that organic carbon supply is not sufficient for sustaining the observed microbial metabolic activity^14^.

Here we present, to the best of our knowledge, the first experimental evidence that deep-sea pelagic viruses actively control prokaryotic population size while sustaining bacterial diversity and community structure. As a consequence, viral lysis could be influencing microbial ecology and biogeochemical cycling in the deep ocean.

## Methods

### Deep-sea water collection and experimental setup

Microcosm experiments were performed onboard the R/V Kexue-1 during the NSFC western Pacific Ocean cruise in December 2010. Samples were collected at a 2000 m depth using 12 L Niskin bottles mounted on a CTD-carousel sampler. Approximately 80 L of deep-sea water was filtered through a 20 μm mesh and 1.0 μm polycarbonate membranes to remove large particles and obtain the fraction containing bacteria and viruses (Fig. 1). Seventy liters of filtrate was filtered through a 0.22 m polypropylene cartridge (Prep-Scale/TFF, Millipore) to obtain the fraction containing viruses. Then, 35 L of filtrate was filtered through a 30 kDa cut-off polysulfone cartridge (Prep-Scale/TFF, Millipore) to obtain virus-free seawater. The bacteria plus virus fractions were diluted with virus-containing and virus-free water, respectively, to ca. 10% of the initial volume to obtain two treatments for deep-sea bacterioplankton: with viruses at an ambient concentration and with diluted viruses (Fig. 1). Two replicates of each treatment were set up in 20 L polycarbonate bottles (Nalgene) and incubated in the dark at the ambient seawater temperature (ca. 2 °C). All bottles and containers were acid-rinsed before use.

### Time series sampling and measurements

Subsamples for determining bacterial and viral abundance were collected twice daily during the incubation and analyzed using an onboard FACSAria flow cytometer (Becton, Dickinson and Company, USA) with SYBR Green I staining following procedures described previously^42,43^. On flow cytometry, particles with high nucleic acids (DNA or RNA) content always show high-fluorescence signal^19,20^. Therefore, the present study considered bacterioplankton with high-fluorescence signal as potentially active bacterial populations. For molecular ecology analysis, 2 L of samples were filtered onto 0.22 μm pore size, 47 mm-diameter polycarbonate filters (Millipore, Bedford, MA, USA) to retrieve the total bacterioplankton population. To start the incubation as soon as possible, we collected bacterial diversity samples before splitting them into +virus and −virus treatments, assuming the bacterial community composition in the treatments at the beginning of the incubation were the same. The filters for DNA analysis were stored at −80 °C, and the samples for RNA analysis were immersed in 600 μL of an RLT solution (Qiagen, Chatsworth, CA, USA) and stored at −80 °C. Ribosomal RNA (rRNA) has been widely employed to indicate potentially active microbial assemblage in various environments (e.g., see refs. ^22,38^). Therefore, we apply bacterial 16S rRNA gene amplicon from environmental DNA and RNA to characterize the whole and potentially active bacterioplankton, respectively, in our study.

### Pyrosequencing and analysis of bacterial diversity

For DNA-based and RNA-based high throughput sequencing of microbial communities, DNA and RNA were extracted using a MoBio PowerWater DNA Isolation Kit (MoBio, San Diego, CA, USA) and an RNeasy Mini Kit (Qiagen, Hilden, Germany), respectively, according to the manufacturers’ protocols. During RNA purification, gDNA contamination was removed using an RNase-Free DNase Set (Qiagen, Hilden, Germany), which was verified by PCR amplification of bacterial 16S rRNA gene. The SuperScript III First-Strand Synthesis System with random hexamers (Invitrogen, Carlsbad, CA, USA) was used to synthesize first-strand cDNA for the RT-PCR. Please note that one RNA extraction of Day 12 sample of −virus treatments was not successful.

Partial 16S rRNA gene fragments were amplified using the universal primers F515 (GTGNCAGCMGCCGCGGTAA) and R926 (CCGYCAATTYMTTTRAGTTT), which cover the V4 and V5 regions of the gene^44^. PCR was carried out in 25 μL reaction volumes with 2× Premix Ex Taq (Takara, Dalian, China) under the following conditions: initial denaturation at 95 °C for 3 min, followed by 30 cycles of 95 °C for 30 s, 55 °C for 45 s, and 72 °C for 45 s, with a final extension at 72 °C for 10 min. Triplicate PCR products for each sample were pooled together and purified using a MiniBEST Agarose Gel DNA Purification kit Ver. 3.0 (Takara). Finally, 454 library preparation and sequencing were conducted at the Shanghai Hanyu Biotechnology Co. (Shanghai, China).

Sequence analysis was carried out using QIIME 1.8.0^45^. The raw sequences were quality filtered for length (300 bp < length < 460 bp), quality score (>25), number of homopolymer runs (<6), and lack of ambiguous bases and ambiguous mismatches in the barcode and primer. Chimeric sequences were removed using ChimeraSlayer^46^. OTUs were identified using UCLUST (version 1.2) at a 97% similarity level, and a representative sequence was selected for further analysis^47^. Taxonomy was assigned using UCLUST with the Greengenes database (version 13.8)^48^. Although the universal primer set 515F-926R was designed for both bacterial and archaeal 16S rRNA genes, we observed a relative low abundance of Archaea sequences (2.87%), which were removed from further analysis using the RDP Classifier^49^. The representative sequences for bacterial OTUs were aligned using PyNAST^50^. OTUs that occurred only once (singletons) were also removed from all eleven libraries. Overall, the analysis yielded a total of 98,939 sequences, and an OTU table was created. To control for variation in the number of sequences between samples, the libraries in the OTU table were normalized to the minimum sample size, resulting in 64,427 sequences in total and 5857 sequences per sample. A phylogenetic tree was constructed using FastTree^51^. All further analyses were performed using the QIIME pipeline based on the OTU table and phylogenetic tree. Meanwhile, the representative sequences of the 58 most abundant OTUs (accounting for 90% of total sequences) were aligned using ARB^52^, and a parsimony tree was constructed using default parameters and a lane mask.

### Network construction

Co-occurrence networks for RNA-inferred bacterial communities were constructed to evaluate whether viruses affected the active interactions among deep-sea bacterioplankton during the 12-day incubation. To ensure the confidence of the network analysis, only the OTUs appearing more than twice (e.g., two time series samples) within each treatment were kept for the following network construction. The correlation network was constructed by the Sparse Correlation for Compositional data algorithm (SparCC) with 20 iterations^53^. The correlation coefficients were averaged over the 20 iterations. In addition, 100 randomly shuffled datasets based on the original datasets were constructed using bootstrap method with replacement. The random networks were also constructed by SparCC with the same parameters used for the practical network construction. Thereafter, we calculated the two-sided pseudo *p* values (*p* values ≤ 0.05 considered significant) for differences between correlations based on practical dataset and those based on 100 randomly shuffled datasets. Then, only correlations between OTUs with values >0.9 and <−0.9 and a pseudo *p* value < 0.05 were presented in the network. The visualization of networks and analysis were performed in Cytoscape 3.5.0^54^. Modules were defined using clusterMaker2, a Cytoscape plugin^55^, with the community clustering algorithm (GLay), which is an implementation of the Girvan–Newman fast greedy algorithm^56^.

### Statistics and reproducibility

Two independent incubations for each treatment were setup onboard. The collection details of samples are described in the “Methods” section. Microbial abundances were determined with two technical replicates by FCM. All the abundance data used for analysis are available in Supplementary Data 1. PCR amplification of bacterial 16S rRNA genes was performed with three technical replicates.

### Reporting summary

Further information on research design is available in the Nature Research Reporting Summary linked to this article.

## Supplementary information

Supplementary Information

Description of Additional Supplementary Files

Supplementary Data 1

Supplementary Data 2

Supplementary Data 3

Reporting Summary

## Data Availability

The entire sequencing data set has been deposited in the NCBI SRA database under project number SRP139068. The data of microbial abundance during microcosm incubation have been provided in Supplementary Data 1. The distribution of major OTUs in each sample is shown Supplementary Data 2. Supplementary Data 3 shows the proportion of nodes belonging to α-Proteobacteria and γ-Proteobacteria in the networks and the proportion of these two classes in the +virus and −virus treatments at RNA level.
