# Supplementary material for: Viral control of biomass and diversity of bacterioplankton in the deep sea
Source: Commun Biol. 2020 May 22;3:256. doi: 10.1038/s42003-020-0974-5 (PMC7244761; doi:10.1038/s42003-020-0974-5)
Supplement: Supplementary file 1 — Supplementary Information [file 42003_2020_974_MOESM1_ESM.pdf]

Supplementary Figure for

Viral control of biomass and diversity of bacterioplankton in the deep sea

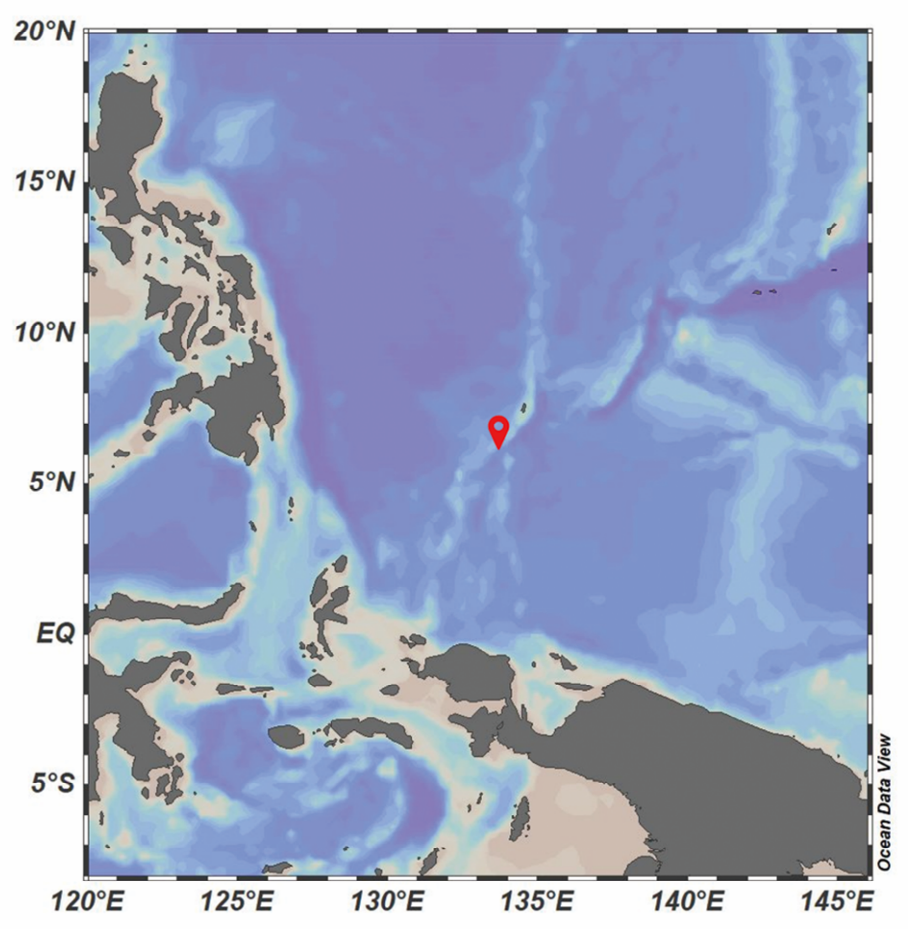

Supplementary Figure 1. Map showing the sampling site. The map was generated using Ocean Data View 4 software (Schlitzer, Reiner, Ocean Data View, odv.awi.de, 2020).

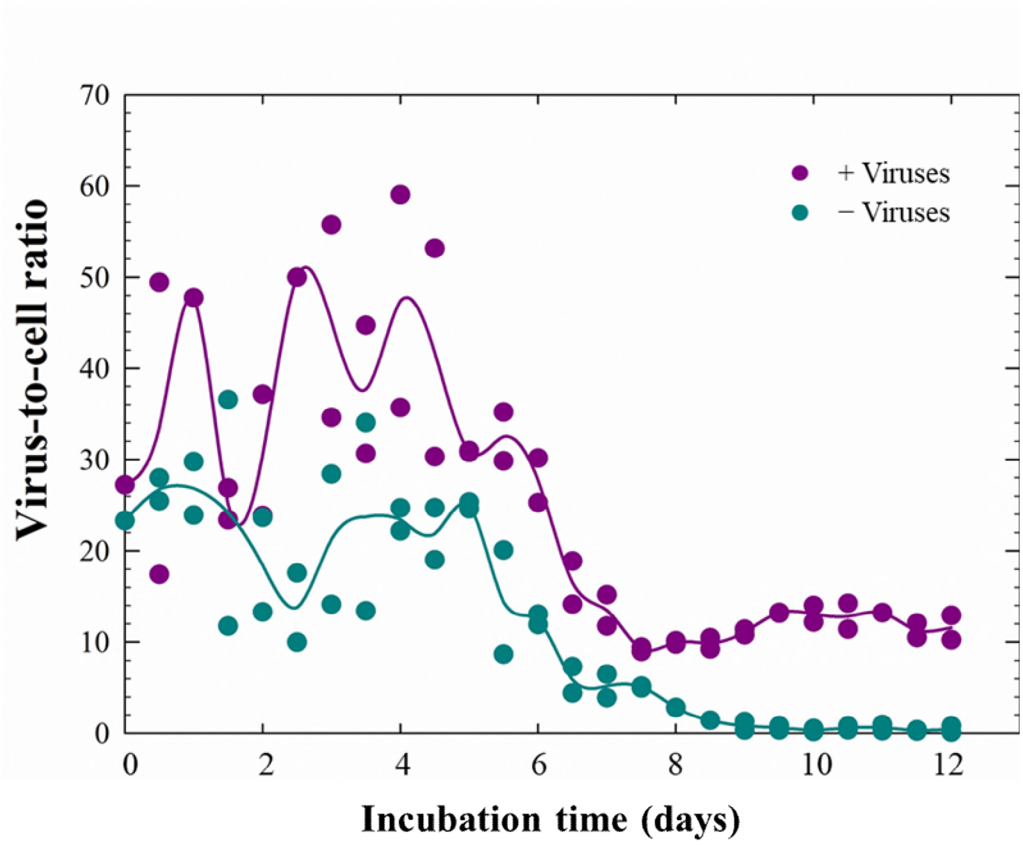

Supplementary Figure 2. Dynamic of virus-to-cell ratio during microcosm incubation.

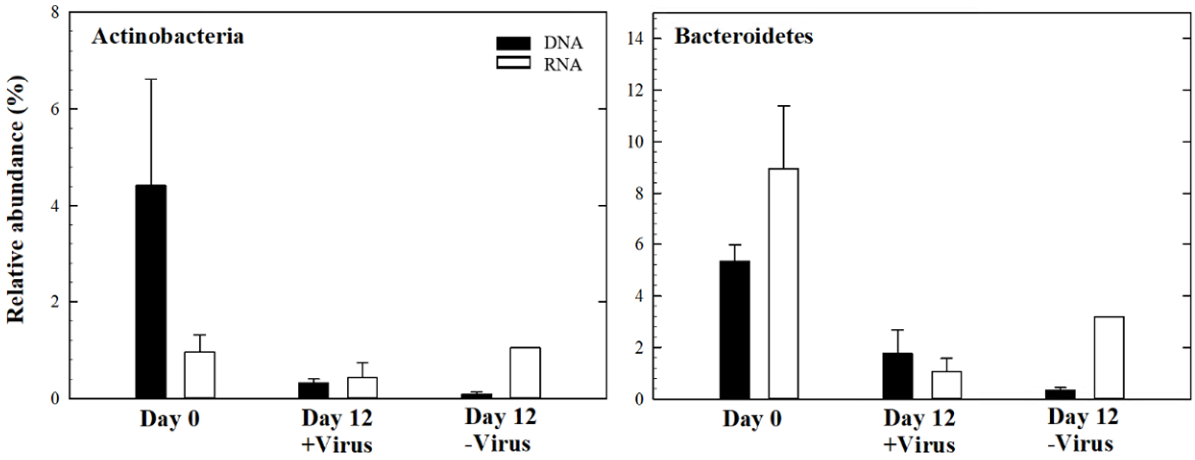

Supplementary Figure 3. Effects of viruses on the relative abundances of total and active Actinobacteria and Bacteroidetes after incubation.

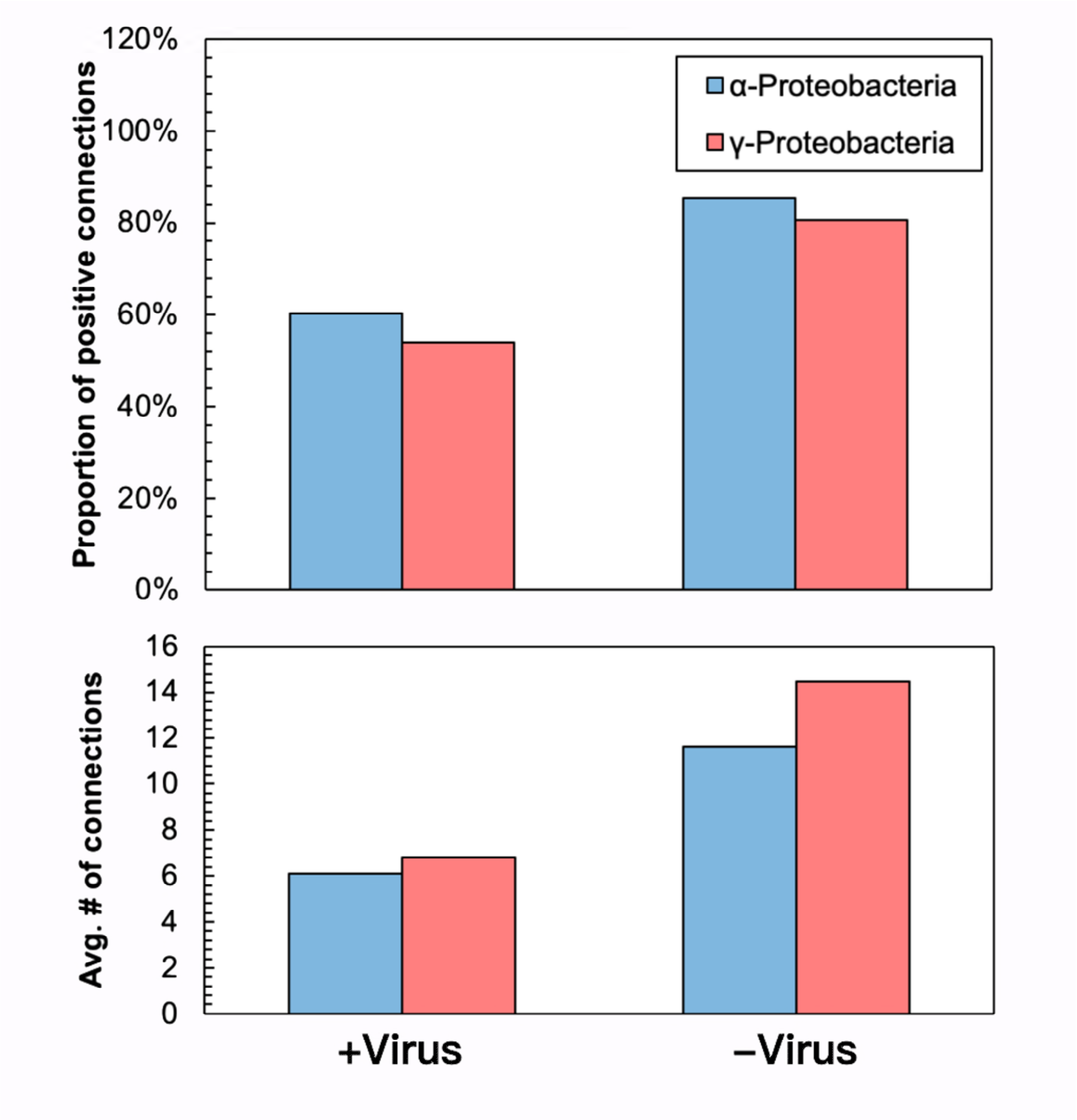

Supplementary Figure 4. Proportion of positive correlations of α-Proteobacteria and γ-Proteobacteria from co-occurrence networks. Lower histogram plot shows the average link number of α-Proteobacteria and γ-Proteobacteria.
