# Supplementary material for: Viral control of biomass and diversity of bacterioplankton in the deep sea
Source: Commun Biol. 2020 May 22;3:256. doi: 10.1038/s42003-020-0974-5 (PMC7244761; doi:10.1038/s42003-020-0974-5)
Supplement: Supplementary file 2 — Description of Additional Supplementary Files [file 42003_2020_974_MOESM2_ESM.pdf]

Supplementary Data legend for

Viral control of biomass and diversity of bacterioplankton in the deep sea

Supplementary Data 1. The data of bacterial abundance, viral abundance, high-fluorescence bacterial abundance and percentages of high-fluorescence bacterial abundance to total bacterial abundance during microcosm incubation. HFBA, high-fluorescence bacterial abundance; TBA, total bacterial abundance.

Supplementary Data 2. The distribution of major OTUs in each sample. The number of the sequences were shown after normalization (5857).

Supplementary Data 3. Proportion of nodes belonging to α- and γ-Proteobacteria in the networks and the proportion of these two classes in the +virus and -virus treatments at RNA level.
