# Supplementary material for: Viral control of biomass and diversity of bacterioplankton in the deep sea
Source: Commun Biol. 2020 May 22;3:256. doi: 10.1038/s42003-020-0974-5 (PMC7244761; doi:10.1038/s42003-020-0974-5)
Supplement: Supplementary file 6 — Reporting Summary [file 42003_2020_974_MOESM6_ESM.pdf]

nature research

Corresponding author(s): Rui Zhang; Lanlan Cai

Last updated by author(s): Feb 22, 2020

Reporting Summary

Nature Research wishes to improve the reproducibility of the work that we publish. This form provides structure for consistency and transparency in reporting. For further information on Nature Research policies, see Authors & Referees and the Editorial Policy Checklist.

Statistics

For all statistical analyses, confirm that the following items are present in the figure legend, table legend, main text, or Methods section.

n/a	Confirmed	
☐	☒	The exact sample size (*n*) for each experimental group/condition, given as a discrete number and unit of measurement
☐	☒	A statement on whether measurements were taken from distinct samples or whether the same sample was measured repeatedly
☐	☒	The statistical test(s) used AND whether they are one- or two-sided<br>*Only common tests should be described solely by name; describe more complex techniques in the Methods section.*
☐	☒	A description of all covariates tested
☐	☒	A description of any assumptions or corrections, such as tests of normality and adjustment for multiple comparisons
☐	☒	A full description of the statistical parameters including central tendency (e.g. means) or other basic estimates (e.g. regression coefficient) AND variation (e.g. standard deviation) or associated estimates of uncertainty (e.g. confidence intervals)
☐	☒	For null hypothesis testing, the test statistic (e.g. *F*, *t*, *r*) with confidence intervals, effect sizes, degrees of freedom and *P* value noted<br>*Give P values as exact values whenever suitable.*
☐	☒	For Bayesian analysis, information on the choice of priors and Markov chain Monte Carlo settings
☐	☒	For hierarchical and complex designs, identification of the appropriate level for tests and full reporting of outcomes
☐	☒	Estimates of effect sizes (e.g. Cohen's *d*, Pearson's *r*), indicating how they were calculated

Our web collection on statistics for biologists contains articles on many of the points above.

Software and code

Policy information about availability of computer code

Data collection	Microcosm experiments were performed onboard the R/V Kexue-1 during the western Pacific Ocean cruise in December 2010. Samples were collected at a 2,000-m depth using 12-L Niskin bottles mounted on a CTD-carousel sampler. Subsamples for determining bacterial and viral abundance were collected twice daily during the incubation and analyzed using an onboard FACSAria flow cytometer (Becton, Dickinson and Company, USA).For molecular ecology analysis, 2 L of water were filtered onto 0.22 µm pore size, 47 mm-diameter polycarbonate filters (Millipore, Bedford, MA, USA). For DNA- and RNA-based high throughput sequencing of microbial communities, DNA and RNA were extracted using a MoBio PowerWater DNA Isolation Kit (MoBio, San Diego, CA, USA) and an RNeasy Mini Kit (Qiagen, Hilden, Germany), respectively. The SuperScript III First-Strand Synthesis System with random hexamers (Invitrogen, Carlsbad, CA, USA) was used to synthesize first-strand cDNA for the RT-PCR.54 library preparation and sequencing were conducted at the Shanghai Hanyu Biotechnology Co. (Shanghai, China).
Data analysis	Sequence analysis was carried out using QIIME 1.8.0. OTUs were identified using UCLUST (version 1.2) at a 97% similarity level.Taxonomy was assigned using UCLUST with the Greengenes database (version 13.8). A phylogenetic tree was constructed using FastTree. The visualization of networks and analysis were performed in Cytoscape 3.5.0.

For manuscripts utilizing custom algorithms or software that are central to the research but not yet described in published literature, software must be made available to editors/reviewers. We strongly encourage code deposition in a community repository (e.g. GitHub). See the Nature Research guidelines for submitting code & software for further information.

Data

Policy information about availability of data

All manuscripts must include a data availability statement. This statement should provide the following information, where applicable:

- Accession codes, unique identifiers, or web links for publicly available datasets
- A list of figures that have associated raw data
- A description of any restrictions on data availability

Accession codes, unique identifiers, or web links for publicly available datasets.

Field-specific reporting

Please select the one below that is the best fit for your research. If you are not sure, read the appropriate sections before making your selection.

☐ Life sciences ☐ Behavioural & social sciences ☒ Ecological, evolutionary & environmental sciences

For a reference copy of the document with all sections, see nature.com/documents/nr-reporting-summary-flat.pdf

Ecological, evolutionary & environmental sciences study design

All studies must disclose on these points even when the disclosure is negative.

Study description	Here we perform a microcosm study for deep-sea bacterioplankton (the hosts of viruses) with and without the pressure of viral lysis in the western Pacific Ocean to demonstrate the impacts of active virioplankton on host communities in deep-sea ecosystems.
Research sample	Deep-sea waters at a 2,000-m depth.
Sampling strategy	Samples were collected using 12-L Niskin bottles mounted on a CTD-carousel sampler onboard the R/V Kexue-1 during the NSFC western Pacific Ocean cruise in December 2010.
Data collection	Samples were collected onboard the R/V Kexue-1 during the NSFC western Pacific Ocean cruise and recorded by Yanxia Li, one of the authors.
Timing and spatial scale	Eighty liters of deep-sea water are collected in December 2010.
Data exclusions	No data were excluded from the analyses.
Reproducibility	Microcosm experiments were performed onboard the R/V Kexue-1 during the western Pacific Ocean cruise in December 2010. Samples were collected at a 2,000-m depth using 12-L Niskin bottles mounted on a CTD-carousel sampler. Subsamples for determining bacterial and viral abundance were collected twice daily during the incubation and analyzed using an onboard FACSAria flow cytometer (Becton, Dickinson and Company, USA).
Randomization	Two replicates of each treatment were set up in 20 L polycarbonate bottles (Nalgene) and incubated in the dark onboard. Subsamples for determining microbial abundance were collected twice daily during the incubation.
Blinding	To ensure the confidence of the network analysis, only the OTUs appearing more than twice (e.g., two time series samples) within each treatment were kept for the following network construction.

Did the study involve field work? ☒ Yes ☐ No

Field work, collection and transport

Field conditions	Sunny
Location	133.7°E, 6.38°N
Access and import/export	Cruise was organized by the National Natural Science Foundation of China in December 2010.
Disturbance	None

Reporting for specific materials, systems and methods

We require information from authors about some types of materials, experimental systems and methods used in many studies. Here, indicate whether each material, system or method listed is relevant to your study. If you are not sure if a list item applies to your research, read the appropriate section before selecting a response.

Materials & experimental systems

n/a	Involved in the study	
☒	☐	Antibodies
☒	☐	Eukaryotic cell lines
☒	☐	Palaeontology
☒	☐	Animals and other organisms
☒	☐	Human research participants
☒	☐	Clinical data

Methods

n/a	Involved in the study	
☒	☐	ChIP-seq
☐	☒	Flow cytometry
☒	☐	MRI-based neuroimaging

Flow Cytometry

Plots

Confirm that:

☒ The axis labels state the marker and fluorochrome used (e.g. CD4-FITC).

☒ The axis scales are clearly visible. Include numbers along axes only for bottom left plot of group (a 'group' is an analysis of identical markers).

☒ All plots are contour plots with outliers or pseudocolor plots.

☒ A numerical value for number of cells or percentage (with statistics) is provided.

Methodology

Sample preparation	Two mL of seawater were fixed with a final concentration of 0.5% glutaraldehyde, incubated at 4 °C for 15 min in the dark, flash frozen in liquid nitrogen, and then stored at -80 °C until analysis. The samples for virus counting were thawed at 37 °C, diluted with Tris-EDTA buffer (pH = 8, Sigma-Aldrich, Darmstadt, Germany), then stained with 5.0 × 10-5 (v/v, final concentration) SYBR Green I (Invitrogen).
Instrument	Flow cytometer (Epics Altra II, Beckman Coulter)
Software	The data acquisition and analysis were performed with the EXPOTM32 MultiCOMP software.
Cell population abundance	The in situ prokaryotic and viral abundances decreased from 0.70 and 6.17 × 106 particles mL-1 in surface waters to 0.49 and 1.70 × 106 particles mL-1 at 2,000 m.
Gating strategy	Microbial particles were identified based on the green fluorescence and side scatter signal using the FCS Express V3 software (De Novo Software).

☒ Tick this box to confirm that a figure exemplifying the gating strategy is provided in the Supplementary Information.
